# Early postoperative recovery in operating room after desflurane anesthesia combined with Bispectral index (BIS) monitoring and warming in lengthy abdominal surgery: a randomized controlled study

**DOI:** 10.1186/s12871-018-0577-6

**Published:** 2018-08-17

**Authors:** Hong Yu, Lu Zhang, Ye Ma, Hai Yu

**Affiliations:** 1 Department of Anesthesiology, West China Hospital of Sichuan University, Chengdu, 610041 People’s Republic of China; 2 Department of Anesthesiology, China-Japan Friendship Hospital, Beijing, 100029 People’s Republic of China

**Keywords:** Anesthesia, General, Anesthetics volatile, Desflurane, Anesthetics volatile, Sevoflurane, Recovery, Anesthesia-controlled time, ACT, Warming, Bispectral index monitoring

## Abstract

**Background:**

This study aimed to determine whether the use of desflurane (DES) anesthesia combined with bispectral index (BIS) monitoring and warming is effective in reducing anesthesia-controlled operating room time (ACT) in patients undergoing lengthy abdominal surgery.

**Methods:**

Seventy patients, 40 years of age or older, undergoing abdominal surgery expected to last three to five hours were randomly assigned to the DES group (*n* = 35) or the control group (n = 35). Patients in the DES group were maintained with desflurane anesthesia and received BIS monitoring and warming. Patients in the control group were given non-desflurane anesthesia for maintenance, and the usage of BIS monitoring and warming were not mandatory and determined by anesthesia care providers. Early postoperative recovery times were recorded.

**Results:**

The times to extubation (8.8 ± 8.5 vs 14.7 ± 13.7 min, *P* = 0.035), eye opening (8.4 ± 8.6 vs 14.4 ± 13.4 min, *P* = 0.028), responds on command (8.2 ± 8.5 vs 14.4 ± 13.0 min, *P* = 0.022), and the ACT (23.8 ± 11.4 vs 32.7 ± 15.4 min, *P* = 0.009) were significantly less in the DES group than that in the control group. The postanesthesia care unit (PACU) length of stay, incidence of prolonged extubation, and surgeon and anesthesiologist satisfaction were similar in two groups. Also, the result of multivariable linear regressions showed that patients who were younger, female, lower BMI and non-DES anesthesia regimen resulted in prolonged extubation.

**Conclusions:**

Desflurane anesthesia combined with BIS monitoring and warming is associated with early postoperative recovery in lengthy abdominal surgery.

**Trial registration:**

ChiCTR-INR-17013333. Date of registration: November 11, 2017.

## Background

Anesthesia-controlled time (ACT) and turnover time are two of the most important factors that regulate operating room (OR) efficiency [1]. Prolonged extubation is an important factor affecting operating room efficiency, which is a concern of surgeons and anesthesia care providers [2, 3]. Prolonged extubation slows work flow and requires OR staff to monitor while waiting for the next operation. Choosing appropriate anesthetic agents or techniques is essential for anesthesia care providers to reduce time of extubation and post-anesthesia recovery.

Desflurane, with the lowest blood-gas partition coefficient of the available halogenated agents, has been suggested be a potential for rapid recovery after discontinuation [4]. Previous meta-analysis found that desflurane reduced the extubation time by 34% relative to isoflurane and by 25% relative to sevoflurane [3, 5]. Compared to propofol, desflurane reduced the mean time to extubation and time to follow commands by 21% and 23%, respectively [2]. However, the time of emergence and extubation between propofol-based total intravenous anesthesia (TIVA) via target-controlled infusion (TCI) system and desflurane are controversial [6].

Besides the use of anesthesia agents, many factors contribute to faster post-anesthesia recovery and extubation. Studies have suggested that intraoperative bispectral index (BIS) monitoring can reduce anesthetic use and recovery times [7, 8]. In addition, inadvertent perioperative hypothermia also impacted recovery quality of general anesthesia by influencing the kinetics and action of various anesthetic and paralyzing agents [9]. The longer anesthesia time, the higher the likelihood the patient would experience hypothermia [10]. Actively warming patients has been demonstrated to offer good conditions for early tracheal extubation [11].

Considering the multiple factors affecting the early extubation, the aim of this study was to explore whether desflurane-based anesthesia combined with BIS monitoring and active warming in lengthy abdominal surgery could reduce the extubation time, improve the quality of post-anesthesia recovery, and satisfaction of surgeons and OR members.

## Methods

### Study design

This study was a prospective randomized controlled trial with two parallel arms undertaken in West China Hospital of Sichuan University, China. The ethics committee of our institution approved the study protocol. All patients provided written informed consent before inclusion. Randomization was performed using a computer-generated randomization sequence and allocation concealment was maintained until the time of anesthesia induction by using opaque, numbered and sealed envelopes. The trial was prospectively registered at Chictr.org.cn (ID ChiCTR-INR-17013333).

### Inclusion and exclusion criteria

Patients were eligible for participation if they met the following criteria: elective abdominal surgery (surgical time > 3 h and < 5 h) and older than 40 years of age. Exclusion criteria were as follows: combined propofol and desflurane anesthesia, combined sevoflurane and desflurane anesthesia, no postanesthesia care unit (PACU) stay, and failure to extubate. Patients who are participating in other interventional studies are also ineligible.

### Experimental protocol

Patients who met the enrollment criteria were randomized 1:1 to either the desflurane (DES) or the control group. General anesthesia was induced with midazolam 0.05 mg kg^− 1^, sufentanil 0.3–0.5 μg·kg^− 1^, propofol 1.5–2 mg kg^− 1^ and cisatracurium 0.15 mg kg^− 1^. The trachea was intubated with an appropriately sized tracheal tube (Mallinckrodt, COVIDIEN, Mexico). Patients were ventilated with the Datex Ohmeda ventilator (Aestiva/5 Compact Plus, Datex-Ohmeda, Freiburg, Germany) by using volume-controlled ventilation and with an inspired oxygen fraction of 0.6 and a peak inspiratory pressure ≤ 30 cmH_2_O. The ventilation rate was adjusted to maintain end-tidal carbon dioxide partial pressure between 35 and 45 mmHg.

In the DES group, anesthesia was maintained with desflurane, continuous infusion of remifentanil 0.1–0.2 μg·kg^− 1^·min^− 1^, and repetitive bolus injection of cisatracurium and sufentanil as required throughout the procedure. Maintenance of the effective desflurane concentrations was adjusted according depth of anesthesia monitored by BIS (Aspect Medical Systems, Newton, MA, USA). Patient temperature was maintained by forced-air warming blanket (setting temperature 38 °C) or warming infusion (setting temperature 37 °C) to avoid nasopharyngeal temperature lower than 36 °C.

In the control group, the only absolute criterion was no desflurane inhalation during the procedure. Anesthesia was maintained with propofol, sevoflurane, or both, and the concentrations were adjusted according BIS or clinical signs. Repeated dose of cisatracurium, sufentanil and continuous infusion of remifentanil 0.1–0.2 μg·kg^− 1^·min^− 1^ were prescribed throughout the procedure. Unlike the DES group, the usage of BIS monitoring and warming were not mandatory and decided by anesthesia care providers.

At the end of the operation, desflurane, sevoflurane or propofol and remifentanil were discontinued, and the lungs were ventilated with 100% oxygen at a fresh gas flow of 8 L/min. Reversal of neuromuscular block was achieved by administrating neostigmine (0.04 mg/kg) with atropine (0.02 mg/kg) once spontaneous breathing returned. After the patient regained consciousness, with spontaneous and smooth respiration, the endotracheal tube was removed and the patient was transferred to the PACU for further care.

### Outcomes

The following times were calculated: 1) extubation time: completion of surgery to extubation; 2) eye opening time: completion of surgery to patients’ eye opening; 3) time to respond on command: completion of surgery to the time when patients can respond on command to squeeze fingers; 4) surgical time: incision to surgical completion and application of dressing; 5) anesthesia time: initiation of anesthesia to extubation; 6) exit from OR time: extubation to exit for OR; 7) total OR time: arrival in the OR to departure from the OR; 8) ACT: the combination of anesthesia induction time, extubation time and exit from OR time; 9) PACU length of stay (LOS): time from arrival in the PACU to meeting the PACU discharge criteria (post anesthesia recovery score ≥ 9 according activity, respiration, circulation, consciousness and color) [12]. Also, the anesthetist and surgeon satisfaction and the incidence of prolonged extubation were recorded. The anesthetist was asked to express the satisfaction with the anesthesia type while the surgeon judged the turnover time of each procedure scored on a scale from 1 to 5 (1 = poor to 5 = excellent). Prolonged extubation is defined as an extubation time equal to or longer than 15 min [5].

### Statistical analysis

We hypothesized that desflurane would cause a 25% reduction of extubation time compared with that in the control group. At 90% power and significance at the two-sided 5% level, this required a sample size of 60 subjects (30 per group), which we increased by 33% to accommodate withdrawal or missing data points. All analyses were performed by an independent expert unaware of the allocated treatment groups.

Data are expressed as mean ± standard deviation (SD) or number (percentage). Comparisons between the two groups were performed using unequal-variance student *t* test for continuous variables. Chi-square or Fisher exact tests were used as appropriate for categorical variable comparisons between groups. Multivariable linear regression analyses were performed to assess the association between variables contributed to prolonged extubation. Results were considered statistically significant at a *P* value less than 0.05. Statistical analyses were performed using statistical software SPSS 17.0.

## Results

### Characteristics of the study population

**Fig. 1:**
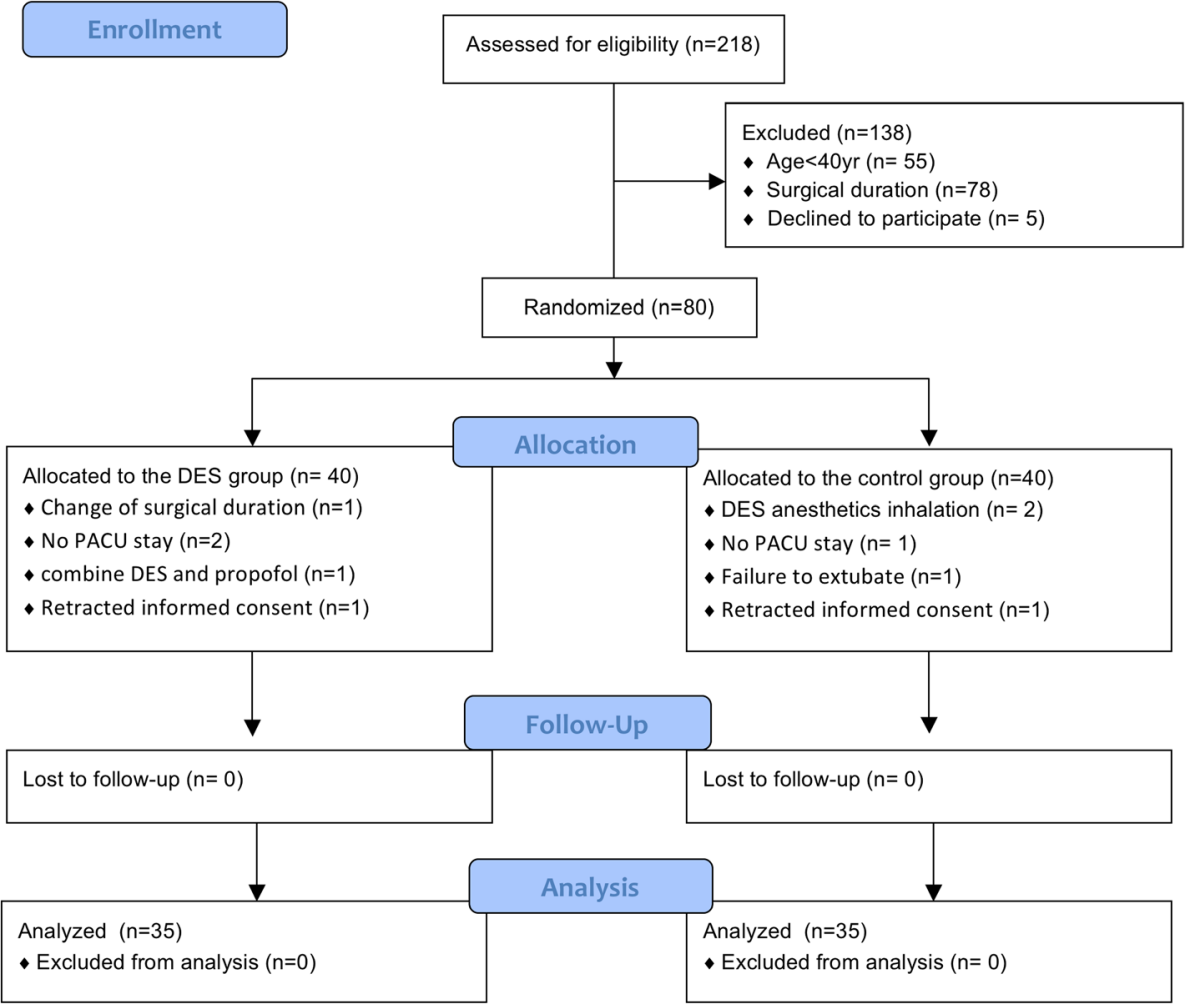
Flow chart

A total of 218 patients undergoing abdominal surgery were recruited. Among them, 80 were included and randomly assigned to the DES group (*n* = 40) or the control group (*n* = 40). After randomization, 10 patients were excluded and 35 patients (DES) vs. 35 patients (control) were included for final analysis (Fig. 1). No significant difference was found between two groups in terms of baseline characteristics. In the control group, sixteen patients received warming, nine received BIS monitoring and two received both (Table 1).

**Table 1:** Baseline patient characteristics

Characteristics	DES (*n* = 35)	Control (*n* = 35)	*P* value
Age (yr)	57.5 ± 10.3	56.2 ± 11.6	0.62
ASA II/III	28/7	27/8	0.77
Gender (M/F)	19/16	24/11	0.22
Body mass index (kg/m^2^)	21.85 ± 2.55	22.68 ± 3.03	0.23
Surgical procedure			
stomach	10 (28.6%)	8 (22.9%)	0.77
liver	11 (31.4%)	15 (42.8%)	0.32
pancreas	3 (8.6%)	5 (14.3%)	0.45
colorectum	11 (31.4%)	7 (20%)	0.27
Anesthesia procedure			
warming	35 (100%)	16 (45.7%)	0.0001
depth of anesthesia monitoring	35 (100%)	9 (20%)	0.0001
both	35 (100%)	2 (5.7%)	0.0001
Agents during anesthesia			
desflurane	35 (100%)	0 (0%)	0.001
TIVA	0 (0%)	9 (26%)	0.001
sevoflurane	0 (0%)	10(28%)	0.001
sevoflurane+propofol	0 (0%)	16 (46%)	0.001
sufentanil, μg	37.8 ± 7.1	38.8 ± 8.9	0.64
remifentanil, μg	1399 ± 441	1466 ± 494	0.55
cisatracurium, mg	23.7 ± 5.1	24.2 ± 6.0	0.71

Dichotomous data are presented as number (%); continuous data are presented as mean (standard deviation). No significant difference was found between the two groups

### Comparison of postoperative recovery between the DES group and the control group

Patients from the DES group were found to have shorter time to extubation, eye opening, and respond on command than patients from the control group (extubation, 8.8 ± 8.5 vs 14.7 ± 13.7 min, *P* = 0.035; eye opening, 8.4 ± 8.6 vs 14.4 ± 13.4 min, *P* = 0.028; responds on command, 8.2 ± 8.5 vs 14.4 ± 13.0 min, *P* = 0.022). Additionally, the ACT in the DES group was significantly shorter than that in the control group (23.8 ± 11.4 vs 32.7 ± 15.4 min, *P* = 0.009). There was no significant difference found in the PACU LOS, incidence of prolonged extubation, and surgeon and anesthetist satisfaction between two groups (Table 2).

**Table 2:** Operating room time measurements between DES and control group

Items	DES Group (*n* = 35)	Control Group (*n* = 35)	*P* value
Extubation time, min	8.8 ± 8.5	14.7 ± 13.7	0.035
Eye opening time, min	8.4 ± 8.6	14.4 ± 13.4	0.028
Time to respond on command, min	8.2 ± 8.5	14.4 ± 13.0	0.022
Anesthesia-controlled time, min	23.8 ± 11.4	32.7 ± 15.4	0.009
Exit From OR after extubation, min	10.6 ± 8.0	11.0 ± 7.3	0.835
Anesthesia induction time, min	6.1 ± 2.4	7.5 ± 4.7	0.132
Surgical time, min	216.2 ± 45.8	221.4 ± 60.2	0.69
Anesthesia time, min	261.2 ± 48.7	262.5 ± 63.2	0.92
Total OR time, min	293.6 ± 50.5	315.5 ± 78.1	0.17
PACU LOS, min	84.0 ± 34.0	92.1 ± 33.4	0.34
Prolonged extubation (≥15 min)	6 (17.1%)	9 (25.7%)	0.38
Anesthetist satisfaction	4.71 ± 0.67	4.54 ± 0.82	0.21
surgeon satisfaction	4.86 ± 0.43	4.71 ± 0.52	0.34

Data are shown as mean ± standard deviation or number (percentage). *DES* desflurane, *OR* operation room, *PACU* postanesthesia care unit, *LOS* length of stay, *SD* standard deviation

### Multivariable linear regressions

The result of multivariable linear regressions comparing prolonged extubation time between several variants in all patients is shown in Table 3. Age, gender, body mass index (BMI) and group were factors that contribute to extubation time. The results showed that patients with younger age, female, lower BMI and non-DES anesthesia regimen have longer extubation time.

**Table 3:** Multivariable linear regression analyses of variables associated with extubation time

	β	95% CI	*P* value
Age	−0.249	(−0.470, −0.028)	0.001
Gender	−6.369	(−11.19, −1.540)	0.028
BMI	−1.063	(−1.947, − 0.178)	0.019
Group	8.541	(3.768, 13.31)	0.001
Surgery time	0.06	(−0.106, 0.226)	0.474
Anesthesia time	−0.026	(−0.183, 0.131)	0.742

β, difference between each variables using extubation time as dependent variable. *Gender* Female 0, Male 1; *Group* The DES group 0, the control group 1; *BMI* Body mass index, *CI* Confidence interval. *P* value< 0.05 were considered significant

## Discussion

The turnover time and ACT are of particular interest to anesthetists and surgeons, and are affected most by anesthetic agents and anesthesia care during surgery. In this randomized trial, we investigated the effects of desflurane-based anesthesia regimen including BIS monitoring and warming on early postoperative recovery in lengthy abdominal surgery. It was observed that desflurane anesthesia regimen when combined with BIS monitoring and warming is associated with reduced extubation time, eye opening time, response on command time, and ACT in lengthy abdominal surgery. However, the PACU LOS was similar between the two groups.

The results of previous studies comparing the extubation time of desflurane with propofol-based TIVA or sevoflurane have been controversial. For the studies comparing the desflurane with sevoflurane, most observed a more rapid recovery from desflurane [4, 13, 14], whereas others comparing desflurane with TIVA found no superiority of desflurane [1, 15, 16]. Sevoflurane and desflurane are both widely used inhalation anesthetics, while desflurane has been shown to be associated with shorter emergence time and earlier recovery time in several studies [14]. Desflurane is characterized by a lower solubility coefficient than sevoflurane, particularly after long–duration surgeries [13, 14, 17]. With regards to the studies comparing desflurane and propofol, a recent meta-analysis found desflurane reduced the extubation time by 21% [2]. However, two observational studies found the use of TIVA with TCI system is more effective than desflurane in reducing ACT [1, 18]. In our study, anesthesia agents for the control group were administered as both propofol or sevoflurane according to the anesthetic care providers, i.e., propofol (9 patients), sevoflurane (10 patients), or combined propofol with sevoflurane (16 patients). Hence, our findings suggest that desflurane anesthesia significantly reduces extubation time (average by 5.9 min or 40%) relative to non-desflurane anesthesia.

One factor to be addressed was that the faster postoperative recovery in the DES group was attributed to not only desflurane but also the BIS monitoring and warming. A recent meta-analysis has shown that the use of monitoring with BIS reduced extubation time, orientation time and PACU LOS [19]. Also, warming to prevent perioperative hypothermia has been reported to reduce the extubation time and improve outcomes in the elderly during lengthy surgery [20]. However, it is worth mentioning that the anesthesia care made by the treating anesthetists in the control group was optional. Therefore, a crossover of treatment between two groups concerning the use of warming and BIS monitoring was observed. In the control group, 66% of patients received at least one of these two measures (i.e. sixteen patients received warming, nine received BIS monitoring and two received both). Consequently, it is rational to assume that the BIS monitoring and warming are widely accepted measures to improve anesthesia care.

The result of multivariable linear regressions showed that patients with younger age, female, lower BMI and non-DES anesthesia regimen resulted in prolonged extubation time, which contradicted previous studies [1, 16, 21, 22]. Lai et al. reported that older age, male, higher BMI, and longer anesthesia time contribute to slower emergence in open major upper abdominal surgery [1]. Chan et al demonstrated that surgical time greater than 210 min and older age, contributed to prolonged extubation for open colorectal surgery [22]. The difference may be caused by sufficiently homogeneity in terms of patient population. First, the patients of Lai and Chan et al. were older than those in our study (68 yrs. and 65 yrs. vs. 57 yrs). Also, we excluded the patients with age younger than 40 years instead of 18 years, and the mosre narrow yet older age range might weaken the relevance between older age and prolonged extubation. Second, the patients’ BMI of Lai and Chan et al. studies were slightly higher than those in our study (24.5 kg/m^2^ and 23.9 kg/m^2^ vs. 22.3 kg/m^2^), although the patients involved were both Asian. In the current study, the average extubation time of eight underweight patients (BMI < 18.5 kg/m^2^, average 17.33 kg/m^2^) were 20 min and that of seven overweight patients (BMI > 25 kg/m^2^, average 26.88 kg/m^2^) were 8 min, and consequently, the multivariable linear regressions showed a negative correlation between BMI and extubation time. Similarly, Suemitsu et al.*.* reported a longer extubation time of underweight patients compared with normal-weight patients (25.3 min vs. 22.7 min) [23]. Lee et al reported the “obesity paradox” applies to colorectal cancer, as indicated by decreased hospital LOS of overweight patients and increased hospital LOS of underweight patients [24]. Meyerhardt et al. demonstrated that weight loss after colorectal cancer-diagnosis was associated with worse cancer-specific mortality and overall mortality [25]. In current study, 64 of 70 (91%) patients were cancer patients and majority of them were complicated with weight loss after cancer-diagnosis. Although it is well known that obesity increases the risk of prolonged extubation, few studies investigated the relationship between being underweight and early recovery in the operating room which further studies are needed. Moreover, we showed that neither surgical time and anesthesia time contribute to prolonged extubation, which might be due to the relative small sample size and restrictive surgery duration of 3 to 5 h.

There are several limitations to the present study. First, the sample size was relatively small, which may cause a potential selection bias. Second, although the data collector and analyzer were blinded, the anesthesia care providers were not blinded. Also, the anesthesia providers involved have different clinical experience levels, which might increase the risk of bias as the proficiency of the anesthesiologist can influence the extubation time. Third, there is the crossover of treatment between two groups. Also, BIS and body temperature were monitored to keep them in the normal range in the DES group only while the data were not recorded or compared between two groups. All of them might underestimate the effect of the desflurane regimen.

## Conclusions

In conclusion, desflurane anesthesia combined with BIS monitoring and warming was shown to be associated with earlier postoperative recovery in lengthy abdominal surgery when compared to non-desflurane anesthesia and standard practice. This can be an alternative used to contribute to faster turnover time and improved OR efficiency in clinical practice.

## Abbreviations

ACT: Anesthesia-controlled time
BIS: Bispectral index
BMI: Body mass index
DES: Desflurane
LOS: Length of stay
OR: Operating room
PACU: Postanesthesia care unit
SD: Standard deviation
TCI: Target-controlled infusion
TIVA: Total intravenous anesthesia

## References

[1] Lai HC, Chan SM, Lu CH, Wong CS, Cherng CH, Wu ZF (2017). Planning for operating room efficiency and faster anesthesia wake-up time in open major upper abdominal surgery. Medicine.

[2] Wachtel RE, Dexter F, Epstein RH, Ledolter J (2011). Meta-analysis of desflurane and propofol average times and variability in times to extubation and following commands. Can J Anaesth.

[3] Dexter F, Bayman EO, Epstein RH (2010). Statistical modeling of average and variability of time to extubation for meta-analysis comparing desflurane to sevoflurane. Anesth Analg.

[4] Gokcek E, Kaydu A, Akdemir MS, Akil F, Akinci IO (2016). Early postoperative recovery after intracranial surgical procedures. Comparison of the effects of sevoflurane and desflurane. Acta cirurgica brasileira.

[5] Agoliati A, Dexter F, Lok J, Masursky D, Sarwar MF, Stuart SB, Bayman EO, Epstein RH (2010). Meta-analysis of average and variability of time to extubation comparing isoflurane with desflurane or isoflurane with sevoflurane. Anesth Analg.

[6] Lai H-C, Chan S-M, Lin B-F, Lin T-C, Huang G-S, Wu Z-F (2015). Analysis of anesthesia-controlled operating room time after propofol-based total intravenous anesthesia compared with desflurane anesthesia in gynecologic laparoscopic surgery: a retrospective study. J Med Sci.

[7] Luginbuhl M, Wuthrich S, Petersen-Felix S, Zbinden AM, Schnider TW (2003). Different benefit of bispectal index (BIS) in desflurane and propofol anesthesia. Acta Anaesthesiol Scand.

[8] Punjasawadwong Y, Phongchiewboon A, Bunchungmongkol N (2014). Bispectral index for improving anaesthetic delivery and postoperative recovery. Cochrane Database Syst Rev.

[9] Doufas AG (2003). Consequences of inadvertent perioperative hypothermia. Best Pract Res Clin Anaesthesiol.

[10] Yi J, Lei Y, Xu S, Si Y, Li S, Xia Z, Shi Y, Gu X, Yu J, Xu G (2017). Intraoperative hypothermia and its clinical outcomes in patients undergoing general anesthesia: national study in China. PLoS One.

[11] Vanni SM, Braz JR, Modolo NS, Amorim RB, Rodrigues GR (2003). Preoperative combined with intraoperative skin-surface warming avoids hypothermia caused by general anesthesia and surgery. J Clin Anesth.

[12] Heavner JE, Kaye AD, Lin BK, King T (2003). Recovery of elderly patients from two or more hours of desflurane or sevoflurane anaesthesia. Br J Anaesth.

[13] Chen G, Zhou Y, Shi Q, Zhou H (2015). Comparison of early recovery and cognitive function after desflurane and sevoflurane anaesthesia in elderly patients: a meta-analysis of randomized controlled trials. J Int Med Res.

[14] Singh PM, Borle A, McGavin J, Trikha A, Sinha A (2017). Comparison of the recovery profile between Desflurane and sevoflurane in patients undergoing bariatric surgery-a meta-analysis of randomized controlled trials. Obes Surg.

[15] Lu CH, Yeh CC, Huang YS, Lee MS, Hsieh CB, Cherng CH, Wu ZF (2014). Hemodynamic and biochemical changes in liver transplantation: a retrospective comparison of desflurane and total intravenous anesthesia by target-controlled infusion under auditory evoked potential guide. Acta Anaesthesiol Taiwanica : Off J Taiwan Soc Anesthesiol.

[16] Wu ZF, Jian GS, Lee MS, Lin C, Chen YF, Chen YW, Huang YS, Cherng CH, Lu CH (2014). An analysis of anesthesia-controlled operating room time after propofol-based total intravenous anesthesia compared with desflurane anesthesia in ophthalmic surgery: a retrospective study. Anesth Analg.

[17] Jakobsson J (2012). Desflurane: a clinical update of a third-generation inhaled anaesthetic. Acta Anaesthesiol Scand.

[18] Lu CH, Wu ZF, Lin BF, Lee MS, Lin C, Huang YS, Huang YH. Faster extubation time with more stable hemodynamics during extubation and shorter total surgical suite time after propofol-based total intravenous anesthesia compared with desflurane anesthesia in lengthy lumbar spine surgery. J Neurosurg Spine. 2015;24(2):268–74. 10.3171/2015.4.SPINE141143 26460755

[19] Oliveira CR, Bernardo WM, Nunes VM (2017). Benefit of general anesthesia monitored by bispectral index compared with monitoring guided only by clinical parameters. systematic review and meta-analysis. Brazilian J Anesthesiol (Elsevier).

[20] Ma H, Lai B, Dong S, Li X, Cui Y, Sun Q, Liu W, Jiang W, Xu F, Lv H (2017). Warming infusion improves perioperative outcomes of elderly patients who underwent bilateral hip replacement. Medicine.

[21] Chan SM, Lee MS, Lu CH, Cherng CH, Huang YS, Yeh CC, Kuo CY, Wu ZF (2015). Confounding factors to predict the awakening effect-site concentration of propofol in target-controlled infusion based on propofol and fentanyl anesthesia. PLoS One.

[22] Chan WH, Lee MS, Lin C, Wu CC, Lai HC, Chan SM, Lu CH, Cherng CH, Wu ZF (2016). Comparison of anesthesia-controlled operating room time between Propofol-based Total intravenous anesthesia and Desflurane anesthesia in open colorectal surgery: a retrospective study. PLoS One.

[23] Suemitsu R, Sakoguchi T, Morikawa K, Yamaguchi M, Tanaka H, Takeo S (2008). Effect of body mass index on perioperative complications in thoracic surgery. Asian Cardiovasc Thorac Ann.

[24] Lee S (2017). The obesity paradox in colorectal Cancer surgery: an analysis of Korean healthcare big data, 2012-2013. Nutr Cancer.

[25] Meyerhardt JA, Kroenke CH, Prado CM, Kwan ML, Castillo A, Weltzien E, Cespedes Feliciano EM, Xiao J, Caan BJ (2017). Association of Weight Change after colorectal Cancer diagnosis and outcomes in the Kaiser Permanente northern California population. Cancer Epidemiol, Biomarkers Prev: Publi Am Assoc Cancer Res, cosponsored by the Am Soc Prev Oncol.

